# Dermoscopy and Skin Cancer

**DOI:** 10.1155/2010/867059

**Published:** 2011-05-02

**Authors:** Iris Zalaudek, Giuseppe Argenziano, Ashfaq A. Marghoob, Giovanni Pellacani, H. Peter Soyer

**Affiliations:** ^1^Department of Dermatology, Medical University of Graz, Auenbruggerplatz 8, 8036 Graz, Austria; ^2^Dermatology Unit, 1st Medical Department, Arcispedale Santa Maria Nuova, 42100 Reggio Emilia, Italy; ^3^Memorial Sloan-Kettering Cancer Center, 1275 York Avenue, New York, NY 10065, USA; ^4^Department of Dermatology, University of Modena and Reggio Emilia, Via del Pozzo, 71-41124 Modena, Italy; ^5^The University of Queensland, St. Lucia, QLD 4067, Australia

When I started my residency training about 11 years ago, I was immediately fascinated by the new morphologic dimension of skin lesions that is offered by dermoscopy, and accordingly, I focused my research on dermoscopy. At that time and as a young researcher, a recurrent statement was that “dermoscopy is not for future research as everything has already been described.” 

Instead, looking back on the research activities in the field of dermoscopy during the last 10 years proves that I was living at the beginning of a new and evolving era of noninvasive, diagnostic imaging techniques. In fact, dermoscopy gained increasing interest over this past decade, which is also reflected by the increasing number of publications on dermoscopy per year. Using, for example, simply “dermoscopy” to search for publications in the ISI Web of Science reveals that the number of publications increased significantly from 17 publications in 2000 to 138 in 2010. As a result, dermoscopy can be considered today as an integrative part in the routine diagnosis and management of patients with skin lesions. 

The fact that even after 10 years of intense research we still discover new features that aid the diagnosis is well reflected by this special issue. In this issue, the dermoscopic features of pigmented intraepidermal carcinoma, CD8-positive solitary pagetoid reticulosis, black hairy tongue, cylindroma, eccrine porocarcinoma, and rippled sebaceoma are described. In addition, a study focusing on the influence of pregnancy on the recurrence of melanoma failed to show an increased risk for recurrence, while another study reports the data of a new transition metal complex for photosensitizing properties and dye-sensitized solar cell.

To this end, I conclude that although dermoscopy in 2011 is no longer a new technique but has become a standard tool for the diagnosis and management of patients with pigmented and nonpigmented skin lesions, there is still need and place for further research in this field.

*Iris Zalaudek* *Iris Zalaudek* *Giuseppe Argenziano* *Giuseppe Argenziano* *Ashfaq A. Marghoob* *Ashfaq A. Marghoob* *Giovanni Pellacani* *Giovanni Pellacani* *H. Peter Soyer* *H. Peter Soyer*

